# New Discovery of Left Atrial Macroreentry Tachycardia: Originating from the Spontaneous Scarring of Left Atrial Anterior Wall

**DOI:** 10.1155/2021/2829070

**Published:** 2021-12-15

**Authors:** Xuefeng Zhu, Hongxia Chu, Jianping Li, Chunxiao Wang, Wenjing Li, Zhen Wang, Zhiyuan Xu, Yanyan Jing, Ruifu Zhao, Lin Zhong, Naibao Hu

**Affiliations:** ^1^Department of Cardiology, Affiliated Yantai Yuhuangding Hospital of Qingdao University, Qingdao University, Yantai, China; ^2^Doppler Ultrasonic Department, Affiliated Yantai Yuhuangding Hospital of Qingdao University, Qingdao University, Yantai, China; ^3^Department of Cardiology, Beijing Anzhen Hospital, Capital Medical University, Beijing, China; ^4^Department of Statistics, Binzhou Medical University, Yantai, China

## Abstract

**Aims:**

This study sought to describe left atrial macroreentry tachycardia (LAMRT) originating from the spontaneous scarring of left atrial anterior wall (LAAW) and its clinical and electrophysiological characteristics, mechanisms, and the formation of substrates.

**Methods and Results:**

9 of 123 patients (89% female, age 79.78 ± 5.59 years) had LAMRT originating from the LAAW with no cardiac surgery or prior left atrial (LA) ablation. The mean tachycardia cycle length (TCL) was 241.67 ± 38.00 milliseconds. Spontaneous scars areas and low voltage areas (LVAs) in the LAAW were found in all patients. Successful ablation of the critical isthmus caused termination of the LAMRT and was not inducible in all patients. Arrhythmogenic substrates of LAMRT were the spontaneous scars of LAAW, which matched with the aorta or/and pulmonary artery contact area. The area under the curve (AUC) of age and combination of gender and age for predicting the LAMRT originating from the LAAW were 0.918 and 0.951, respectively, with a cutoff value of ≥73.5 years of age and gender (female) predicting LAMRT with 88.9% sensitivity and 89% specificity.

**Conclusion:**

Combination of gender and age provides a simple and useful criterion to distinguish LAMRT from cavotricuspid isthmus- (CTI-) dependent atrial tachycardia in macroreentry atrial tachycardia (MRAT) in patients without a history of surgery or ablation. Aorta or/and pulmonary artery contacting LA may be related to spontaneous scars. Ablation the isthmus eliminated LAMRT in all patients.

## 1. Introduction

Left atrial macroreentry tachycardia (LAMRT) is observed most frequently in patients who have undergone prior cardiac surgery or catheter ablation (CA) for atrial fibrillation (AF) [1–6]. However, there is subgroup of patients presenting with primary LAMRT associated with a spontaneous left atrial anterior wall (LAAW) scar, without any prior cardiac interventions [7–12]. Epidemiology, the characteristics, and the formation of arrhythmogenic substrate of this macroreentrant atrial tachycardia (MRAT) are incompletely understood. The aims of this study were (1) to describe the clinical and electrophysiological characteristics of the LAMRT in patients without a history of cardiac interventions, (2) to investigate the characteristics of arrhythmogenic substrate, (3) to figure out the cause of scar formation on the LAAW, and (4) to guide clinicians to predict and prevent this particular type of arrhythmia.

## 2. Methods

### 2.1. Study Population

In this study, we retrospectively evaluated 123 patients with MRAT who were referred to our center for CA from October 2018 to October 2020. Nine patients of LAMRT without obvious structural heart disease and no history of previous surgical or catheter intervention were included. Seven patients underwent enhanced cardiac computed tomography (CT) of left atrium (LA) and 2 patients underwent transesophageal echocardiography (TEE) to exclude LA thrombus. The current study's protocol was approved by the Yantai Yuhuangding Hospital (Yantai, China) Ethics Committee (registration number: 2017-203). All patients gave written informed consent prior to ablation.

### 2.2. Electrophysiological Study, Electroanatomic Mapping of the Atria, and Catheter Ablation

All procedures were performed under conscious sedation and local anaesthesia. The details of the electrophysiological study (EPS), electroanatomic mapping, and catheter ablation procedures are described in Supplementary Material online, Supplemental Method 1. The low voltage areas (LVAs) were defined as the presence of a bipolar voltage amplitude ≤0.45 mV. Scar was defined as no active record or no electrical area with bipolar voltage amplitude ≤0.10 mV. The percentage of LVAs was expressed as the sum of areas with bipolar voltage ≤0.45 mV divided by total LA surface area.

### 2.3. Merge the Voltage Map and the Enhanced Cardiac CT

In order to clarify the cause of LAAW scar formation, we merged the 3D electroanatomic voltage maps and enhanced cardiac CT images to observe the anatomical relationship between LAAW scar and aorta and pulmonary artery, respectively, for 7 patients. One patient's mapping data of tachycardia was lost. The anatomical distance between the LA and those structures was measured, and any anterior external structures of <3 mm in distance from the LA anterior wall were defined as having contact. Subsequently, we traced the contact areas inside the LA on each 2-3 mm CT slice, and 4 areas were created on the 3D maps.

### 2.4. Follow-Up

All patients were monitored at the hospital for at least 24 hours after ablation. After ablation, antiarrhythmic drug therapy was discontinued in all patients. Patients were followed up in the outpatient clinic 1 month and 3 months after surgery and every 3 months after surgery. Holter monitoring was performed at 6 and 12 months and beyond as dictated by symptoms. Recurrence was defined by any documented episode of atrial tachycardia lasting >30 s.

### 2.5. Statistical Analysis

Continuous variables were expressed as mean ± SD, and noncontinuous variables as proportions (percentages). Continuous variables with normal distribution were compared using Student's *t* test. Continuous variables with nonnormal distribution were compared using the Mann–Whitney *U* test. Categorical variables were compared using the chi‐square test or Fisher's exact test. To determine the association among variable indicators and LAMRT, the multivariate linear regression analyses and Pearson correlation analyses were performed. We performed receiver operating characteristic (ROC) curve analysis for prediction of the LAMRT. The SPSS 22.0 software (SPSS Inc., Chicago, IL, USA) was used for data analysis. A value of *P* < 0.05 was considered to indicate statistical significance.

## 3. Results

### 3.1. Patient Characteristics

This study included 123 patients (46 females; age 67.5 ± 11.9 years) with MRAT; 100 patients underwent CA of right atrial tachycardia (83 patients (18 female; age 64.86 ± 9.71 years) with cavotricuspid isthmus- (CTI-) dependent atrial tachycardia and without a history of surgery or ablation, 17 patients with postsurgical intervention). Of the remaining 23 patients who underwent CA of LAMRT, a total of 9 patients (8 females; age 79.78 ± 5.59 years) had no history of previous surgical or catheter intervention. Two of 9 patients (22.2%) had a history of AF. Eight patients (88.9%) had long-term history of hypertension (11.78 ± 8.14 years) and 7 patients (77.8%) had mild to moderate pulmonary hypertension (≥25 mmHg) (1 patient had a history of pulmonary embolism, and 2 patients had a history of chronic obstructive pulmonary disease). Well-controlled hypertension was present in 5 (62.5%) patients. To further characterize the cause of the spontaneous scar of this unusual type of LAMRT, the patient characteristics were also analyzed in 16 of 83 patients (5 females; age 59.06 ± 8.78 years) with CTI-dependent atrial tachycardia and AF (9 patients of paroxysmal AF and 7 patients of persistent AF who have no LVAs in LAAW) who underwent PV isolation within the same period. Bipolar voltage maps were created during distal CS pacing in this group. Baseline characteristics of 2 groups are listed in Table 1. No procedure-related complications were observed.

### 3.2. Characteristics of the Surface ECG P Wave

In all patients, P waves in V1 were completely or predominantly positive, and lead I and aVL showed low amplitude negative or flat. The P wave had an isoelectric interval between adjacent waves which was observed in all patients (Supplementary material: Surface ECG of patients).

### 3.3. Characteristics of Arrhythmogenic Substrate and Reentrant Circuits

**Figure 1:**
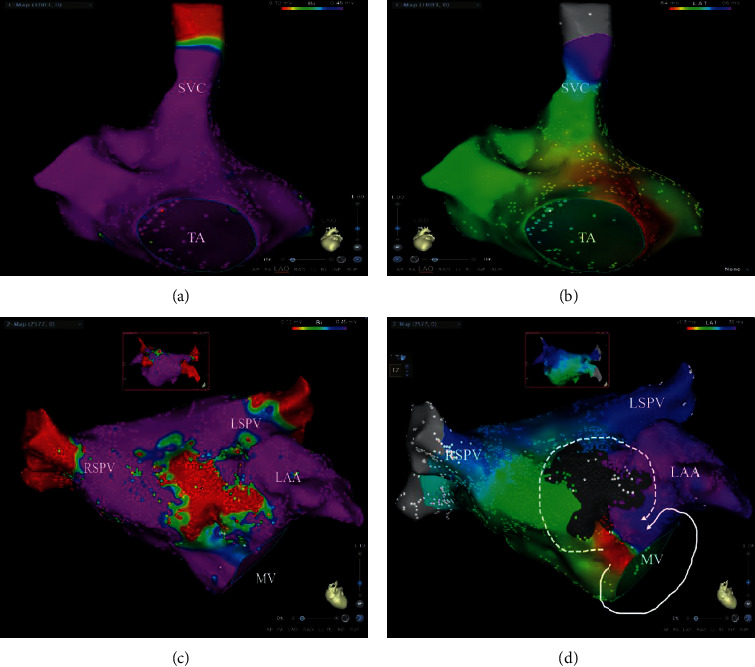
Left atrial macroreentry tachycardia promoted by anterior scar. (a) The low voltage areas were not found in RA. (b) A nonreentrant activation with the earliest site in the atrial septum and the total activation time in the RA < 90% of the TCL. (c) Bipolar voltage map of the left atrium in anterior-posterior and posterior-anterior wall (insets). A central scar was seen in left atrial anterior wall (LAAW). (d) Left atrial activation maps of the patients with a double-loop reentry, a counterclockwise loop around the mitral valve (white solid lines), and a clockwise loop (white dotted lines) around the scar of LAAW. SVC: superior vena cava; TA: tricuspid annulus; LAA: left atrial appendage; MV: mitral valve; RSPV: right superior pulmonary vein; LAAW: left atrial anterior wall.

**Figure 2:**
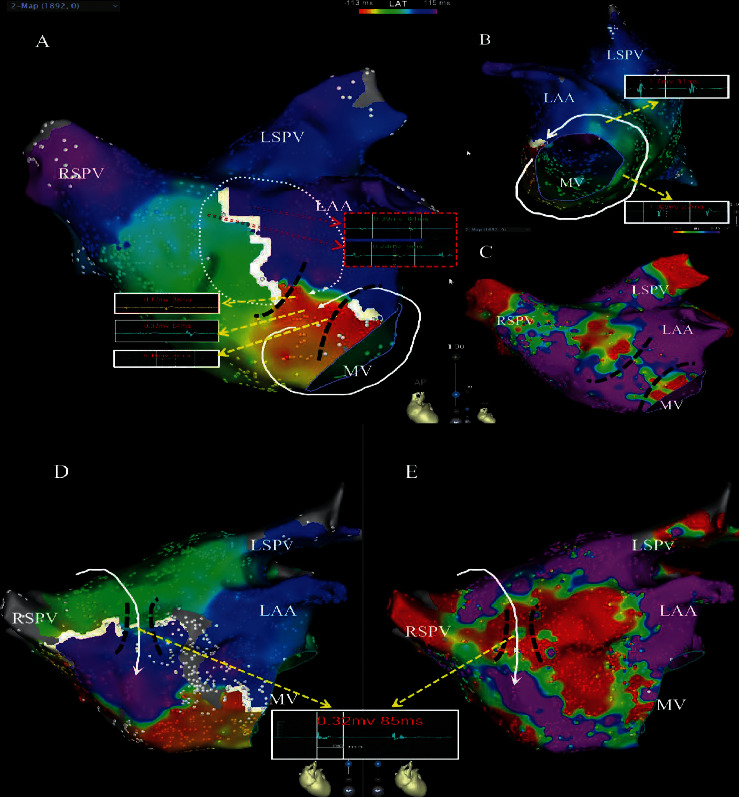
The electrophysiological characteristics of arrhythmogenic substrate. Left atrial activation maps of a patient (a–c) with a double-loop reentry by a critical isthmus (a, c, black dotted lines), which is shown through early meet late in CARTO 3 V6 (action time of more than 25 percent of the TCL between adjacent points indicates block lines (a, red boxes)). The electrograms and conduction velocities of isthmus are shown and selected for ablation (a, white boxes). In contrast, the conventional anatomical isthmus located at the mitral isthmus exhibited normal electrogram characteristics (b, white boxes). The voltage maps (purple ≥ 0.45 mV; red: <0.1 mV) characterized by a central scar at the mid anterior level (c, e). A patient presented with single-loop reentry, and a clockwise loop revolved around the right pulmonary vein (d, e). The isthmus (black dotted lines) located between LAAW scar and the roof adjacent to the RSPV. Tachycardia was terminated in 10 seconds by ablation of the isthmus, where it showed a conduction slowing highly fractionated electrograms (d, e, white boxes) (see Supplementary Material Video 1,2). LAA: left atrial appendage; MV: mitral valve; LSPV: left superior pulmonary vein; RSPV: right superior pulmonary vein; RPV: right pulmonary vein; LAAW: left atrial anterior wall.

**Figure 3:**
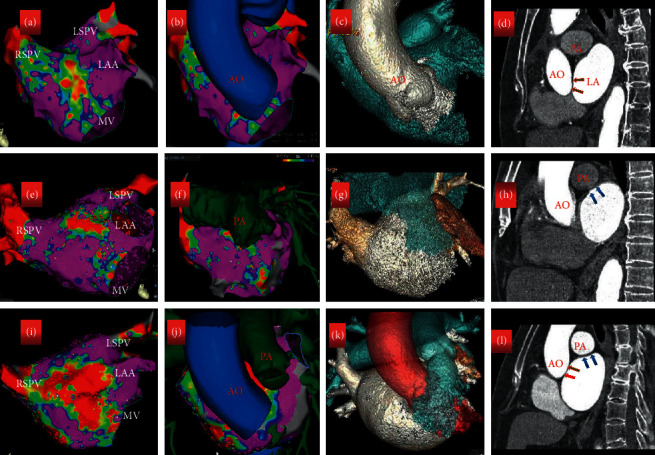
Fusion of the voltage map and the enhanced cardiac CT. Low-voltage area on the LAAW at the left atrium (LA)-aorta (Ao) (a–d) or LA-pulmonary artery (PA) (e–h) or both (i–l) contiguity. The three-dimensional (3D) electroanatomic voltage maps in anteroposterior views (a, e, i) and merged (b, f, j) with 3D enhanced cardiac computed tomography (CT) (c, g, k) corresponding images of enhanced cardiac CT (d, h, l). Ao:  aorta; LA: left atrium; PA: pulmonary artery; LAA: left atrial appendage; MV: mitral valve; LSPV: left superior pulmonary vein; RSPV: right superior pulmonary vein.

**Figure 4:**
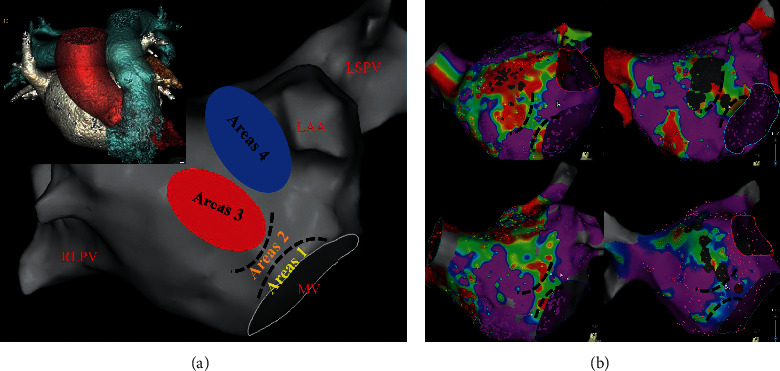
Subsegmentation of the low-voltage area on the left atrial anterior wall. (a) Area 1 was near the anterior MV at 11-12 o'clock; area 2 is the critical isthmus; area 3 was located at the mid anterior level of the left atrial anterior wall (LAAW); area 4 was located at the anterior top of the left atrium (LA) near the basal of left atrial appendage (LAA). (Top left illustration) the three-dimensional (3D) enhanced cardiac computed tomography shows the anatomical relationship between the LA, aorta, and pulmonary artery. (b) Voltage maps show patients with different degrees of scarring have a critical isthmus (black dotted lines) between the scar of LAAW and the MV. LAA: left atrial appendage; MV: mitral valve; LSPV: left superior pulmonary vein; RSPV: right superior pulmonary vein.

Mapping and ablation characteristics of LAMRT are provided in Table 2. The mapping data was lost for some reason in 1 patient. High-density maps were created in 7 patients. In 1 patient, the electroanatomical map was acquired by manual point annotation still allowing accurate identification of scar pattern and mechanism of MRAT. The mean TCL was 241.67 ± 38.00 milliseconds (range 190–310 milliseconds). Mapping of the RA and LA was performed with 1061.25 ± 891.97 points (range: 237–2581 points)/1935.875 ± 682.966 points (range: 660–2808 points) to reconstruct the RA and LA. LVAs and scars were not found to be involved in RA but were found in LAAW in all patients (Figures 1–4). Eight of 9 patients presented with double-loop reentry, a counterclockwise loop around the mitral valve (MV) and a clockwise loop around the LAAW scar (Figures 1 and 2(a)–2(c), Supplementary Material Video 1). The 2 loops shared a common isthmus (7.51 ± 2.27 mm in length and 9.59 ± 2.38 mm in width). The narrow isthmus is located between LAAW scar and the anterior MV in voltage maps except in 4 patients, where it was located between LAAW scar and the basal left atrial appendage (LAA). The mean conduction velocity (CV) of the isthmus was reduced (0.19 ± 0.06 m/s). One of 9 patients presented with single-loop reentry, a clockwise loop revolved around the right PV (Figures 2(d) and 2(e), Supplementary Material Video 2). Low voltage amplitude (0.16 ± 0.05 mv, range: 0.08–0.24 mv), long duration (120.86 ± 26.30 milliseconds, range: 86–159 milliseconds), and fractionated electrograms were found in the isthmus (Figure 2). The percentage of low voltage was 26.58 ± 8.18% (range: 17.1–37%). Interestingly, no LVA was seen in the posterior wall in any of the patients in our study.

### 3.4. Ablation Targets and Ablation Strategies

Seven patients of the tachycardia were terminated with linear ablation, when connecting the MV to the central scar, who were terminated only by 1–9 CA applications across the isthmus within a mean time of 49.7 ± 16.5 seconds (Supplementary Material Video 1). In 1 patient, the clinical tachycardia was converted to another atrial tachycardia after 2 CA applications at the isthmus between LAAW scar and the basal LAA, which was shown to be a focal tachycardia in the posterior wall of the LA adjacent to the right superior pulmonary vein (RSPV) ostium and was successfully ablated. In the single-loop reentry LAMRT of patient, the tachycardia was terminated by 1 CA application at the isthmus between LAAW scar and the roof adjacent to the RSPV ostium, where there were ablation areas of slow conduction and highly fractionated electrograms (Figures 2(d) and 2(e), Supplementary Material Video 2). Because 2 patients had history of AF, the PVI was performed, and an additional line was extended between the scar area and the RSPV. After ablation, the original MRAT could not be induced by programmed atrial stimulation in all the patients. Details of all mapped LAMRT for all patients are shown in Table 3.

### 3.5. Relationship between Anatomy, Gender, Age, and LAAW Scar Formation

**Figure 5:**
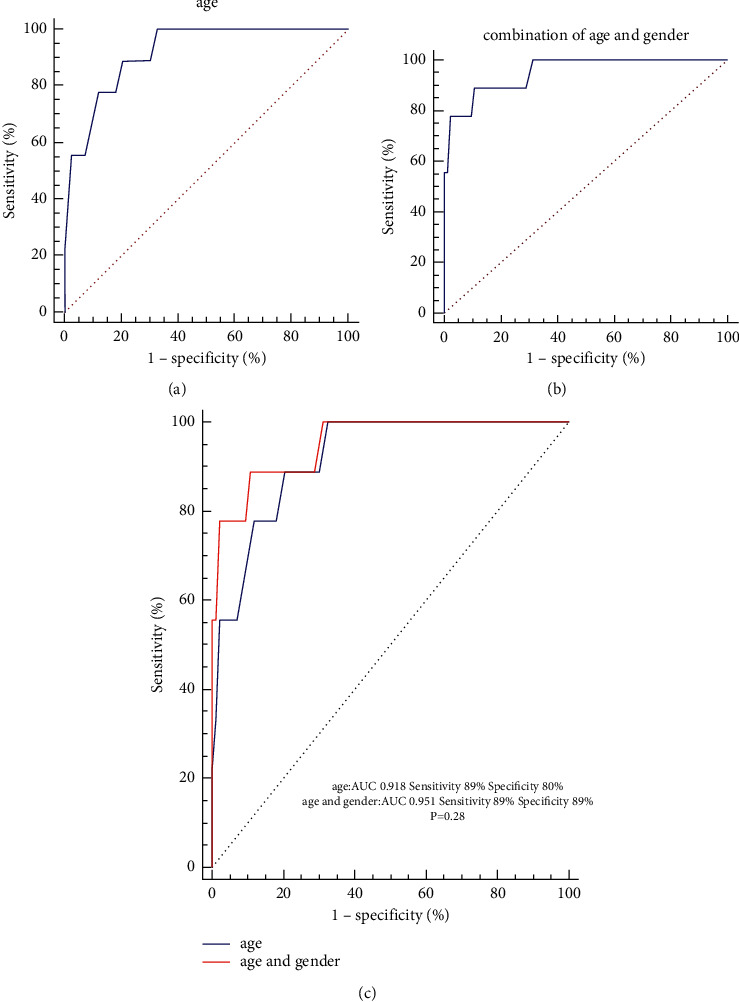
The receiver operating characteristic (ROC) analysis of age and combination of gender and age for predicting the LAMRT originating from the LAAW. The area under the curve (AUC) of age and combination of gender (and age for predicting the LAMRT originating from the LAAW) were 0.918 (a) and 0.951 (b), respectively. (c) Although the comparison between the two was not statistically significant, the combination of gender and age showed a higher specificity. LAAW: left atrial anterior wall; LAMRT: left atrial macroreentry tachycardia.

The 3D mapping of LA and CT images of LA was merged with Carto 3-based substrate maps obtained during MRAT. The LVA of LAAW was subdivided into four areas for detailed analysis: area 1 was near the anterior MV at 11-12 o'clock, which is due to the MV-aorta junction; area 2 is the critical isthmus; area 3 was located at the mid anterior level of the LAAW, which is the contact area between the sinus of Valsalvas and LA; areas 4 was located at the anterior top of the LA near the basal of LAA, which is the contact area between the pulmonary artery and the LA, which were observed in CT slices that they are in contact with each other (Figures 3 and 4). We found that both overall and CTI-dependent atrial tachycardia in MRAT patients were more common in males, but LAMRT was more common in elderly female patients with no previous surgical history with chronic hypertension and/or pulmonary hypertension (Table 4). The multivariate logistic regression of the variables with an unadjusted *P* < 0.05 showed that only gender and age were independent predictors of LAMRT originating from the LAAW (*P*=0.037 and *P*=0.034, resp.) (Table 5). The entire area under the curve (AUC) produced by ROC curve analysis is shown in Figure 5. The area under the curve (AUC) of age and combination of gender and age for predicting the LAMRT originating from the LAAW were 0.918 (Figure 5(a) and 0.951 (Figure 5(b)), respectively, with a cutoff value of ≥73.5 years of age and gender predicting LAMRT with 88.9% sensitivity and 89% specificity (Figure 5(c)).

### 3.6. Clinical Outcomes

Termination of the clinical MRAT could be achieved in all patients. After a mean follow-up of 351 ± 181 days, arrhythmia recurred in 2 patients 4 days and 2 months after surgery. The ECG findings were not the same as before. However, the patient did not undergo ablation again due to old age and economic reasons. All other patients are in stable sinus rhythm without antiarrhythmic drug therapy.

## 4. Discussion

In this study, we systematically investigated a series of patients with primary scar-related LAMRT. The main findings of this study are as follows: (1) we found that arrhythmogenic substrates of LAMRT were the spontaneous scars of LAAW. (2) The critical isthmus is usually located between the LAAW scar and the anterior MV or RPV. (3) A novel finding of this study is that the LVA of LAAW has a consistency between the LA and the aorta or LA and pulmonary artery contiguous area. (4) Moreover, the combination of gender and age can effectively predict this particular type of MRAT. CA linear lesions between the scar of the LAAW and the MV or RSPV seem to be an effective and safe therapy for this arrhythmia.

### 4.1. Arrhythmogenic Substrates and Reentry Circuits

In this study, the arrhythmogenic substrates of this unusual type of MRAT were revealed by high-density mapping. The LVA and spontaneous scar were consistently presented and located in LAAW. The predominantly observed mechanism of MRAT could be related to a central anterior scar that appears to form an isthmus between the scar of LAAW and the MV or RPV. The CV in the isthmus was slower than that in other parts of the heart. On the whole, the isthmus showed the following obvious characteristics such as abnormal local electrogram duration, low signal voltage, fractionation, and conduction slowing, which is critical for stabilizing such circuits.

### 4.2. Previous Studies of Pathogenesis of the LA Scarring

In this study, low voltage was found in all patients and only in the LAAW, which was unexpected and impressive. Fukamizu et al. described 6 patients with LAMRT originating from the spontaneous scars of LAAW [10]. Schaeffer et al. described 15 patients who had LAMRT and no history of LA ablation or cardiac surgery. The LVA of LAAW was predominantly related to 8 patients by formation of the isthmus between the LVA and the MV [12]. Mueller-Edenborn et al. reported that LVA is unevenly distributed in the LA but most frequently involves the anteroseptal LA [13]. The above study is very similar to our studies. Kishima et al. demonstrated that the existence of LVA of LAAW was associated with higher LA stiffness index [14]. Nakatani et al. demonstrated that a thin LA wall is an independent predictor of LVA in patients with paroxysmal AF [15], but in the study the septal wall was thinner than all other walls, so the septal wall LVA was more extensive than the LVAs of roof, posterior, and bottom walls. The results are not consistent with what we found which focuses on LAAW. A recently reported phenomenon called “fibrotic atrial cardiomyopathy (ACM)” was responsible for some of the atrial arrhythmias, including AF, atrial tachycardia, or sick sinus syndrome [16]. However, in our study, all the biatrial voltage mapping show a LVA only in the LAAW, which contributed to the MRAT substrates, whereas the right atrium voltage map was normal. At the same time, during our follow-up after ablation, none of the patients showed sick sinus syndrome except for 2 patients with recurrence, so this explanation is also hard to be convincing.

### 4.3. Our Study of Pathogenesis of the LA Scarring

Why is LVA only present in the LAAW? Hori Y et al. reported that external structures have an LA anatomical contact area, where there were frequent sites of LVA and fractionated electrograms in patients [17]. Pak et al. demonstrated that the LAAW corresponds to the LVA in the contiguous aorta-LA area around the MV in the 11-12 o'clock direction [18]. Our study has shown different findings. We also found that the LVA is not only partially consistent with the aorta, but also partially consistent with the pulmonary artery by merging enhanced CT and Carto 3 mapping. As from the CT images, the scar of LAAW could represent areas of direct contact regions from the aorta or/and the pulmonary artery. However, to the best of our knowledge, no other report has described the anatomic associations of the LVZ of the LAAW in patients with scar-related LAMRT. Scarring around the anterior wall of the MV (area 1) is due to at the MV-aorta junction, and the LA is continuous through the subaortic curtain with the musculature of the anterior mitral leaflet. This region can generate abnormal electrical activity [19], but the exact mechanism of voltage reduction in the contiguous aorta-LA or pulmonary artery-LA area is unclear. Age, gender, hypertension, pulmonary hypertension, and left atrial enlargement may contribute to the formation of LAAW scar. The morphologic enlargement of the LAAW is more extensive in the setting of LA dilation [20]. We also found that MRAT patients were mostly older female with a long history of hypertension and mild to moderate pulmonary hypertension in addition to left atrial dilatation, which can cause dilation of the aorta and pulmonary artery, which can lead to closer contact between them, and its contact against the constantly pulsating aorta and pulmonary artery may result in a LVA on LAAW, although we found no significant difference in aortic and pulmonary artery diameters between the study group and control group. Chronic hypertension can lead to impaired left ventricular diastolic function, resulting in increased left ventricular pressure, which increases left atrial pressure and volume, more often encountered in women with AF than in men. Aging has been shown to be associated with regional conduction slowing and structural changes that include areas of low voltage [21]. In postmenopausal women, the increased sympathetic tone [22, 23], the pronounced decrease in estrogen [24], the increase in epicardial fat and metabolic syndrome [25], and increased diastolic dysfunction [26–28] may contribute to the formation of atrial fibrosis. As women age, their atrial functional decay is more severe [29]. However, the detailed relationship between sex, age, the external structures, and arrhythmogenic substrates is still unclear and requires further investigation.

### 4.4. Clinical Implications for Future Therapies and Research

Although the typical MRAT (CTI-dependent flutter) is most common in patients, the LAMRT mostly occurs in elderly female patients with hypertension or pulmonary hypertension for many years. If we clinically encountered elderly female MRAT patients with chronic hypertension and pulmonary hypertension without any prior cardiac interventions, we should first consider this type of MRAT [30]. The tachycardia could be eliminated with 1–9 RF by selecting the critical isthmus with low signal amplitude, long duration, and fractionated electrograms in the majority of LAMRT. The critical isthmus is usually located between the LAAW scar and the anterior MV or RPV, which is clinical guiding significance for novices or electrophysiologists who are unable to perform high-density mapping. During CA for AF, if a LVA is found on the LAAW of the patient with no history of MRAT, the ablation line should be routinely performed between an LVA and an anatomic obstacle (the MV or/and RPV) to prevent this type of MRAT.

### 4.5. Study Limitations

This study has several limitations: (1) the patients included in this retrospective study were a highly selected group referred for CA, and the number of patients was also limited. (2) It is found that the LVA is anatomically consistent with the aorta or pulmonary artery, and the detailed relationship between the external structures and arrhythmogenic substrates is still unclear and requires further investigation, which need more detailed mechanistic studies. (3) Voltage maps were acquired during MRAT mapping, and we did not perform additional voltage mapping in sinus rhythm. (4) Are these patients most elderly female, because our sample size is insufficient, or are elderly females at high risk? We need to expand the sample size and find out what is special about it. (5) Although this study showed that the combination of gender and age can effectively predict this particular type of MRAT, further prospective studies are needed to confirm its predictive value.

## 5. Conclusions

We demonstrated that extensive spontaneous LAAW scarring is an unusual cause of LAMRT in patients without obvious structural heart disease or previous surgery or catheter intervention, which is more common in elderly females with a history of hypertension. The LVA of LAAW has a consistency between the LA and the aorta or LA and pulmonary artery contiguous area. RF catheter ablation of the critical isthmus could be successfully ablated and eliminate the LAMRT.

## Figures and Tables

**Table 1:** Patient characteristics.

Patients characteristics	Study group (*N* = 9)	Control group (*N* = 16)	*P* value
Age (yrs)	79.78 ± 5.59	59.06 ± 8.78	≤0.001
Females, *n* (%)	8 (88.9)	5 (31.3)	0.006
Body mass index (kg/m^2^)	23.04 ± 1/798	24.11 ± 1.69	0.151
Hypertension, *n* (%)	8 (88.9)	6 (37.5)	0.013
History of hypertension (yrs)	11.78 ± 8.136	2 ± 3.3559	≤0.001
Diabetes, *n* (%)	1 (11.1)	2 (12.5)	0.918
Coronary artery disease, *n* (%)	3 (33.3)	3 (18.8)	0.412
Chronic obstructive pulmonary disease, *n* (%)	2 (22.2)	0 (0)	0.049
Pulmonary embolus disease, *n* (%)	1 (11.1)	0	0.174
Echocardiography			
Mean LA size (mm)	43.0 ± 4.33	40.27 ± 5.82	0.175
Mean RV size (mm)	22.56 ± 2.603	24.06 ± 2.98	0.218
Mean LV size (mm)	41.0 ± 5.123	45.25 ± 4.34	0.038
Mean IVS size (mm)	10.67 ± 1.803	10.81 ± 1.424	0.825
Mean LVEF (%)	60.78 ± 9.72	65.75 ± 5.94	0.124
Sinus of Valsalva diameter (mm)	29.33 ± 2.87	30.31 ± 4.045	0.529
Aortic annular diameter (mm)	19.33 ± 2.062	20.38 ± 2.062	0.238
Ascending aorta diameter (mm)	32.22 ± 4.494	32.88 ± 4.646	0.736
Pulmonary artery diameter (mm)	24.00 ± 3.00	22.94 ± 1.879	0.285
Pulmonary artery pressure (mmHg)	28.33 ± 8.26	18.25 ± 8.347	0.009
BNP (pg/mL)	364.162 ± 287.03	119.634 ± 168.128	0.039

Values are mean ± SD, *n* (%), or mean (range). LA: left atrium; RV: right ventricular; LV: left ventricular; IVS: interventricular septum; LVEF: left ventricular ejection fraction; BNP: B-type natnuretic peptide.

**Table 2:** Mapping and ablation characteristics of tachycardia.

Procedure duration (min)	121.2 ± 35.4
Fluoroscopy time (min)	11.2 ± 5.4
Fluoroscopy dose (cGycm2)	131.5 ± 57.6
High-density mapping performed	7
Mapping points per RA map, *n*	1061.25 ± 891.97
Mapping points per LA map, *n*	1935.875 ± 682.966
TCL (ms)	241.67 ± 38.00
Double-loop reentry atrial tachycardia	8 (89%)
Critical isthmus (*n* = 9)	
Between the LAAW scar and the anterior mitral annulus	7
Between LAAW scar and the base of the LAA	1
Between LAAW scar and the roof adjacent to the RSPV	1
Isthmus width (mm)	9.59 ± 2.38
Isthmus length (mm)	7.51 ± 2.27
Percentage of (%) LVA (≤0.45 mV)	26.58 ± 8.18
Anatomic relationship with LVA (*n* = 6)	
Ascending aorta	1
Pulmonary artery	3
Ascending aorta and pulmonary artery	2
Overall tachycardia termination	9
To sinus rhythm	8
To secondary atrial tachycardia	1

Values are mean ± SD, *n* (%), or mean (range). TCL: tachycardia cycle length; RA: right atrium; LA: left atrium; LAAW: left atrial anterior wall; LAA: left atrial appendage; RSPV: right superior pulmonary vein; LVA: low voltage area.

**Table 3:** Mapping data of atrial tachycardia.

Patient	Gender	Age	AF history	TCL (ms)	Mapping points per RA map, *n*	Mapping points per LA map, *n*	Percentage of low-voltage areas (≤0.45 mV)	At circuit	LAAW activation	MV activation	Avg CV at isthmus (m/s)	Amplitude at isthmus (mV)	Durationat isthmus (ms)	Ablation approach	Tachycardia termination	PVI performed	Follow-up
1	F	84	Yes	220	1547	2001	36	Double loop: isthmus between an LAAW scar and the MV	CW	CCW	0.18	0.11	87	Scar to MV	Yes	Yes	Sinus
2	F	86	No	210	153 (no HD-map)	2577	23.7	Double loop: isthmus between an LAAW scar and the MV	CW	CCW	0.20	0.24	132	Scar to MV	Yes	No	Sinus
3	F	77	No	235	1003	1888	21.6	Double loop: isthmus between an LAAW scar and the MV	CW	CCW	0.22	0.20	159	Scar to MV	Yes	No	Recurrence
4	F	82	No	310	(Incomplete map)	1311	19	Double loop: isthmus between an LAAW scar and the MV	CW	CCW	0.19	0.18	121	Scar to MV	Yes	No	Recurrence
5	M	74	Yes	290	2581	2808	17.1	Double loop: isthmus between an LAAW and the base of the LAA	CW	CCW	0.06	0.16	127	Scar to MVand scar to LAA	No to localizedatrial tachycardia	Yes	Sinus
6	F	70	No	190	1359	2116	37	Double loop: isthmus between an LAAW and the MV	CW	CCW	0.21	0.08	134	Scar to MV	Yes	No	Sinus
7	F	77	No	225	237 (no HD-map)	660 (no HD-map)	—	Double loop: isthmus between an LAAW and the MV	CW	CCW	—	—	—	Scar to MV	Yes	No	Sinus
8	F	82	No	250	1610	2126	31.5	Single loop: isthmus between an LAAW and the RSPV	Surround the right pulmonary veins	0.25	0.17	86	Scar to RSPV	Yes	No	Sinus	
9	F	86	No	245	—	—	—	Double loop: isthmus between an LAAW and the MV	—	—	—	—	Scar to MV	Yes	No	Sinus	

ATatrial tachycardia; AF: atrial fibrillation; CV: conduction velocity; *F*: female; M: Male; LA: left atria; MV: mitral valve; PVI: pulmonary vein isolation; RA: right atria; CCW: counterclockwise; CW: clockwise; LAAW: left atrial anterior wall; TCL: tachycardia cycle length; HD: High-density; RSPV: right superior pulmonary vein.

**Table 4:** Patient characteristics.

Patients characteristics	Study group (*N* = 9)	Control group (*N* = 83)	*P* value
Age (yrs)	79.78 ± 5.59	64.86 ± 9.71	≤0.001
Females, *n* (%)	8 (88.9)	18 (21.7)	≤0.001
Body mass index (kg/m^2^)	23.04 ± 1.798	23.52 ± 1.97	0.49
History of AF, *n* (%)	2 (22.2)	18 (21.7)	0.97
Hypertension, *n* (%)	8 (88.9)	43 (51.8)	0.034
Diabetes, *n* (%)	1 (11.1)	18 (21.7%)	0.46
Coronary artery disease, *n* (%)	3 (33.3)	12 (14,5)	0.145
Pulmonary hypertension, *n* (%)	7 (77.8%)	20 (24.1)	0.001
Echocardiography			
Mean LA size (mm)	43.0 ± 4.33	40.38 ± 5.74	0.19
Mean IVS size (mm)	11.67 ± 2.06	11.12 ± 2.05	0.45
Mean RV size (mm)	22.33 ± 2.45	23.16 ± 3.18	0.454
Mean LV size (mm)	41.0 ± 5.123	46.24 ± 7.13	0.035
Mean LVEF (%)	60.78 ± 9.72	59.65 ± 9.85	0.745
Sinus of Valsalva diameter (mm)	29.33 ± 2.87	30.77 ± 3.64	0.256
Aortic annular diameter (mm)	19.33 ± 2.062	20.24 ± 2.22	0.244
Ascending aorta diameter (mm)	32.22 ± 4.494	32.60 ± 3.71	0.775
Pulmonary artery diameter (mm)	24.00 ± 3.00	23.67 ± 6.78	0.883
Blood tests			
Albumin (g/L)	38.00 ± 3.91	39.20 ± 3.77	0.36
TC (mmol/L)	3.90 ± 0.51	4.51 ± 1.07	0.008
LDL-c (mmol/L)	2.26 ± 0.48	2.78 ± 0.92	0.099
HDL-c (mmol/L)	1.14 ± 0.30	1.26 ± 0.33	0.288
Triglyceride (mmol/L)	1.32 ± 0.35	1.18 ± 0.64	0.509
Fasting blood glucose (mmol/L)	6.25 ± 1.50	6.20 ± 2.17	0.948
BNP (pg/mL)	343.43 ± 273.36	245.00 ± 473.66	0.543
D-dimer (mg/L)	1.22 ± 1.77	0.75 ± 0.76	0.447
Serum creatine (*μ*mol/L)	74.89 ± 16.91	88.08 ± 102.04	0.701
Blood urea nitrogen (mmol/L)	7.45 ± 2.03	7.02 ± 5.05	0.803
Uric acid (mmol/L)	388.22 ± 102.46	380.13 ± 114.46	0.839
Homocysteine (*μ*mol/L)	17.92 ± 9.06	14.25 ± 5.15	0.065
ALT (*μ*/L)	19.78 ± 6.34	34.06 ± 71.65	0.553
AST (*μ*/L)	22.11 ± 3.26	45.65 ± 183.33	0.705
WBC (∗10^9^/L)	6.99 ± 2.13	6.62 ± 1.63	0.527
Hemoglobin (g/L)	137.78 ± 11.14	148.58 ± 17.44	0.073
Platelet (∗10^9^/L)	228.44 ± 35.51	217.35 ± 52.55	0.539
Medication use, *n* (%)			
ACEI or ARB,	4 (44.4)	27 (32.5)	0.473
B-blocker	6 (66.7)	37 (44.6)	0.207
Calcium-channel blocker	6 (66.7)	34 (41.0)	0.140
Amiodarone	4 (44.4)	25 (30.1)	0.380

Values are mean ± SD, *n* (%), or mean (range). AF: atrial fibrillation; LA: left atrium; RV: right ventricular; LV: left ventricular; IVS: interventricular septum; LVEF: left ventricular ejection fraction; TC: total cholesterol; LDL-c: low-density lipoprotein cholesterol; HDL-c: high-density lipoprotein cholesterol; BNP: B-type natriuretic peptide; ALT: alanine transaminase; AST: aspartate transaminase; WBC: white blood cell count; ACEI: angiotensin-converting enzyme inhibitor; ARB: angiotensin receptor blockers.

**Table 5:** Multivariate logistic regression analyses of multiple variables and the LAMRT originating from the LAAW.

Variables	OR	95% CI	*P* value
Gender	0.046	0.003–0.835	0.037
Age	1.251	1.018–1.538	0.034
Hypertension	0.647	0.028–14.841	0.786
Pulmonary hypertension	5.239	0.265–103.477	0.277
TC	0.844	0.327–2.176	0.725
LV	0.916	0.725–1.157	0.460

TC: total cholesterol; LV: left ventricular.

## Data Availability

The datasets generated during the current study are not publicly available yet, due to privacy concerns and ongoing additional research. Data can be made available for peer review on reasonable request through contacting the corresponding author.

## Abbreviations

LAAW: Left atrial anterior wall
TCL: Tachycardia cycle length
LAMRT: Left atrial macroreentry tachycardia
AF: Atrial fibrillation
MRAT: Macroreentry atrial tachycardia
CA: Catheter ablation
LA: Left atrial
LVA: Low-voltage area
CT: Computed tomography
RA: Right atrial
PV: Pulmonary vein
SD: Standard deviation
ROC: Receiver operating characteristic curve
AUC: Area under the receiver operating characteristic curve
CTI: Cavotricuspid isthmus
MV: Mitral valve
LAA: Left atrial appendage
CV: The mean conduction velocity.

## Supplementary Materials

Supplementary Materials The Supplementary Material include Supplemental Method 1, Surface ECG of patients, Video 1, and Video 2.
