# Supplementary material for: New Discovery of Left Atrial Macroreentry Tachycardia: Originating from the Spontaneous Scarring of Left Atrial Anterior Wall
Source: J Interv Cardiol. 2021 Dec 15;2021:2829070. doi: 10.1155/2021/2829070 (PMC8694995; doi:10.1155/2021/2829070)

**Supplementary Appendix**

This appendix has been provided by the authors to provide readers with additional information about the study.

Supplement to:  Xuefeng Zhu, Hongxia Chu, Jianping Li et al. New discovery of left atrial macroreentry tachycardia: originating from the spontaneous scarring of left atrial anterior wall

**TABLE OF CONTENTS**

**Page 3-Supplemental Method 1. Electrophysiological study, electro-anatomic mapping of the atria and catheter ablation.**

**Page 5-Surface ECG of patients.**

**Electrophysiological Study**

During the procedure, patients were studied under conscious sedation with continuous intravenous fentanyl. The catheters were introduced through the left subclavian and right femoral vein. A 6-French decapolar deflectable catheter (Biosense Webster, Diamond Bar, CA) was positioned into the coronary sinus (CS). A 5-spline multi-electrode catheter(Pentaray, Biosense Webster, Diamond Bar, CA) was used for mapping. Generally, all patients initially performed right atrial (RA) mapping even though P wave morphology or the activation sequence of the CS catheter suggested the LA origin of the MRAT. If post-pacing interval (PPI)–tachycardia cycle length (TCL)>20 ms or nonreentrant activation with the earliest site in the atrial septum indicated that the RA was not likely involved in the tachycardia circuit, the LA activation mapping should be performed through transseptal approach. If the patient was in sinus rhythm at the start of the procedure, the arrhythmia was induced using atrial programmed stimulation or burst pacing. The clinical MRAT was assumed when cycle length and p-wave morphology matched the clinical documentation of the arrhythmia.

**Electroanatomic Mapping**

Electroanatomical mapping and catheter ablation procedures were performed in all patients using a 3-dimensional(3D) electroanatomical mapping system(CARTO 3, Biosense Webster). A stable signal from the CS was selected as the time reference. During the stable tachycardia, the bipolar voltage and activation mapping were performed (semi-) automatically based on 5 criteria, which consisted of catheter motion, timing stability between 2 reference electrodes (coronary sinus electrograms), cycle length stability, respiration gating, and tracking quality. After completing the electroanatomic map, the wave front propagation and activation patterns were analyzed to further characterize the tachycardia. The low voltage areas (LVAs) were defined as the presence of a bipolar voltage amplitude ≤0.45 mV. Scar was defined as no active record or no electrical area with bipolar voltage amplitude ≤0.10 mV. The percentage of LVAs was expressed as the sum of areas with bipolar voltage ≤0.45 mV divided by total LA surface area.

**Catheter Ablation**

After the tachycardia circuit was mapped, the critical isthmus with low signal amplitude, long-duration, and fractionated electrograms was selected for ablation. The ablation line was performed between a scar area and an anatomic obstacle. This line was extended to the pulmonary vein (PV) if an electrical silent anchor point was absent but substantial regional conduction slowing within the isthmus was present. CA applications were performed with power-control mode, the power and temperature limited to 35-40W and 43°C, and saline irrigation (17 to 30 mL/min) and with a 3.5-mm-tip open-irrigated contact force-sensing catheter (ThermoCool Smart Touch, Biosense Webster, South Diamond Bar, CA). Procedure success was defined as termination and an inability to induce tachycardia.

**Surface ECG of patients**


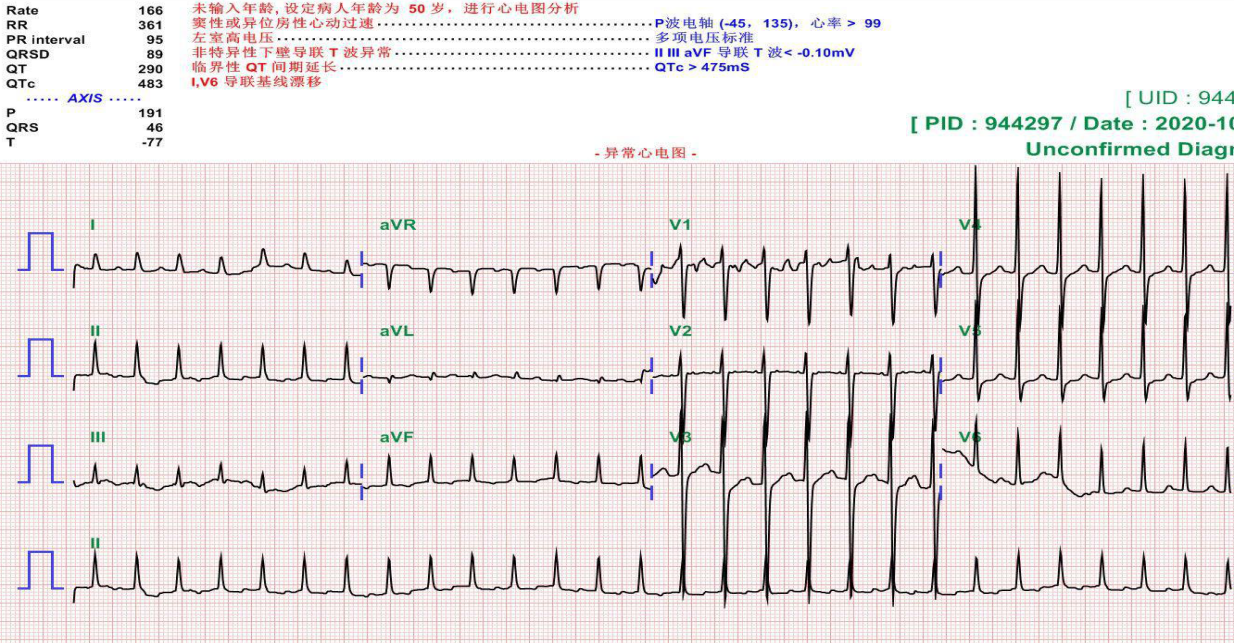

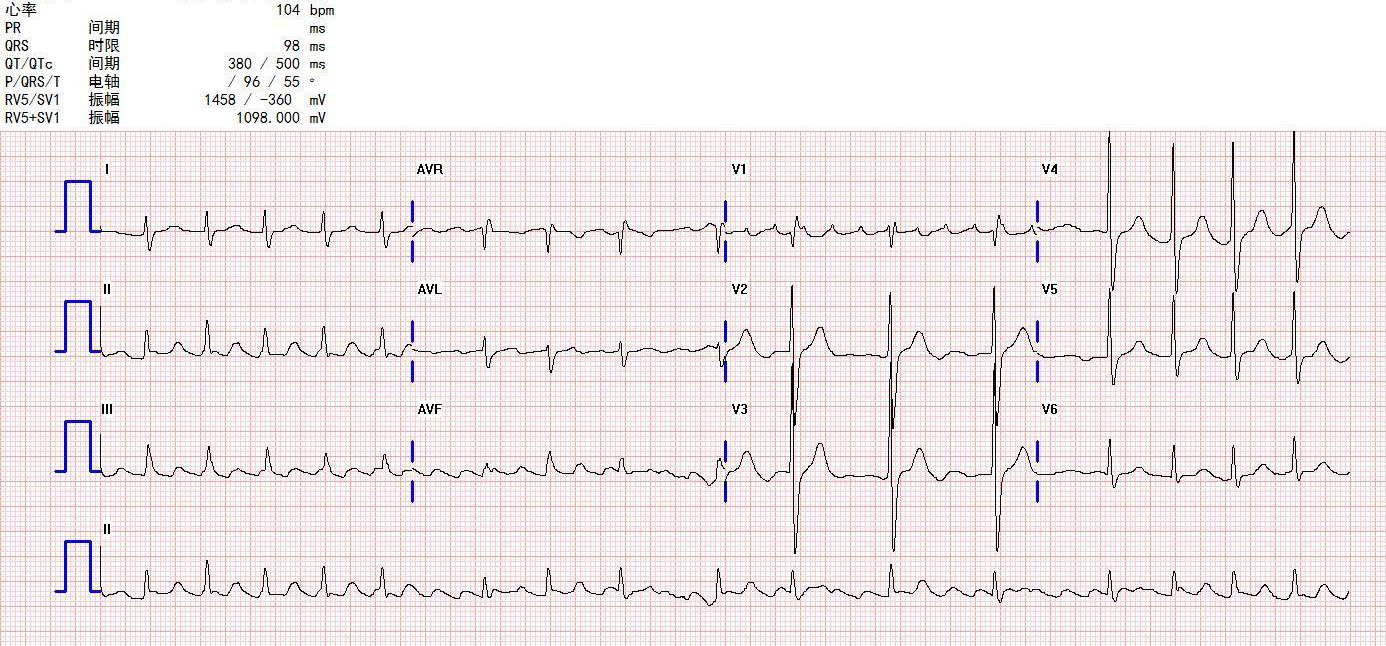

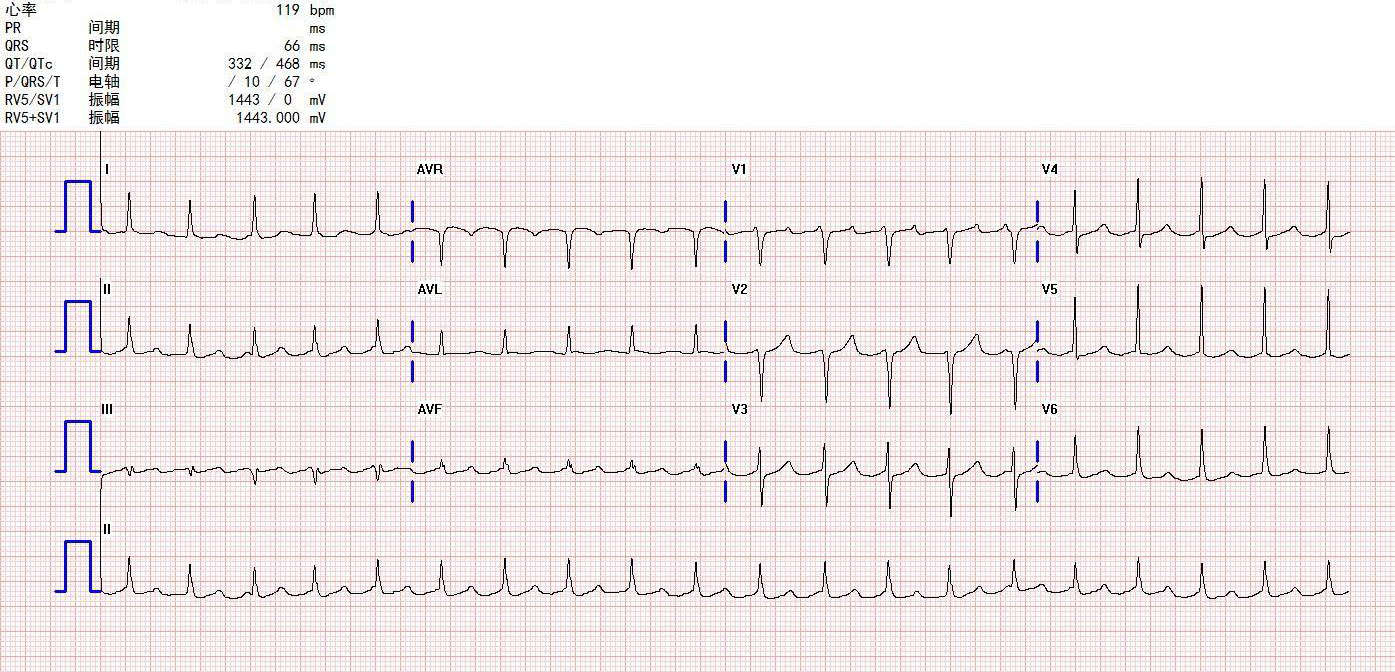


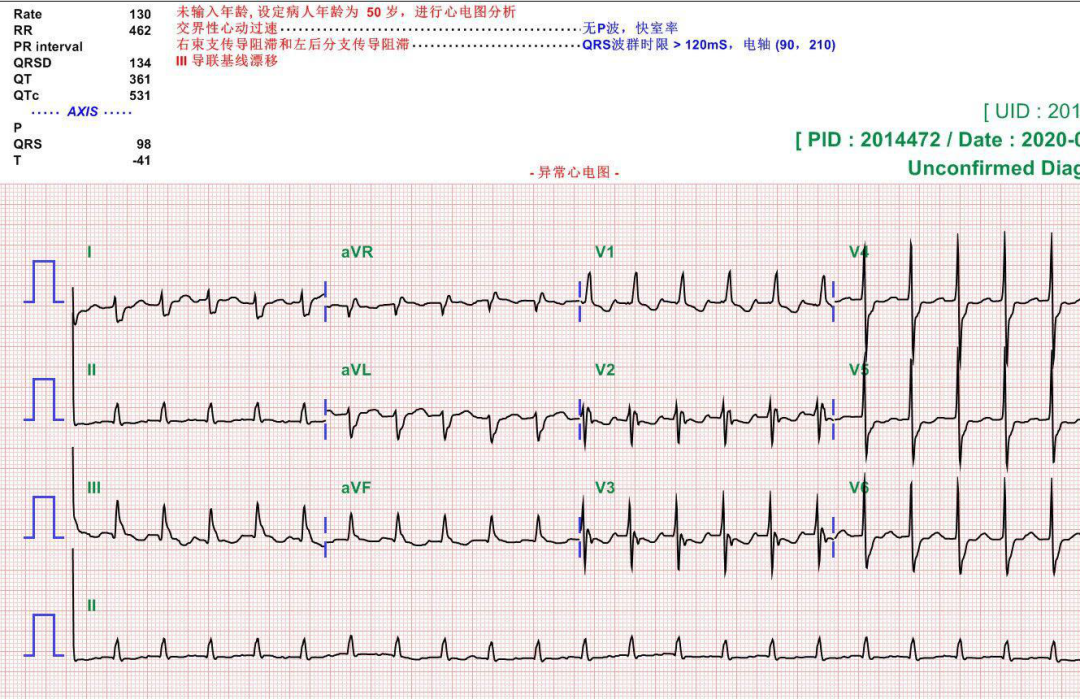


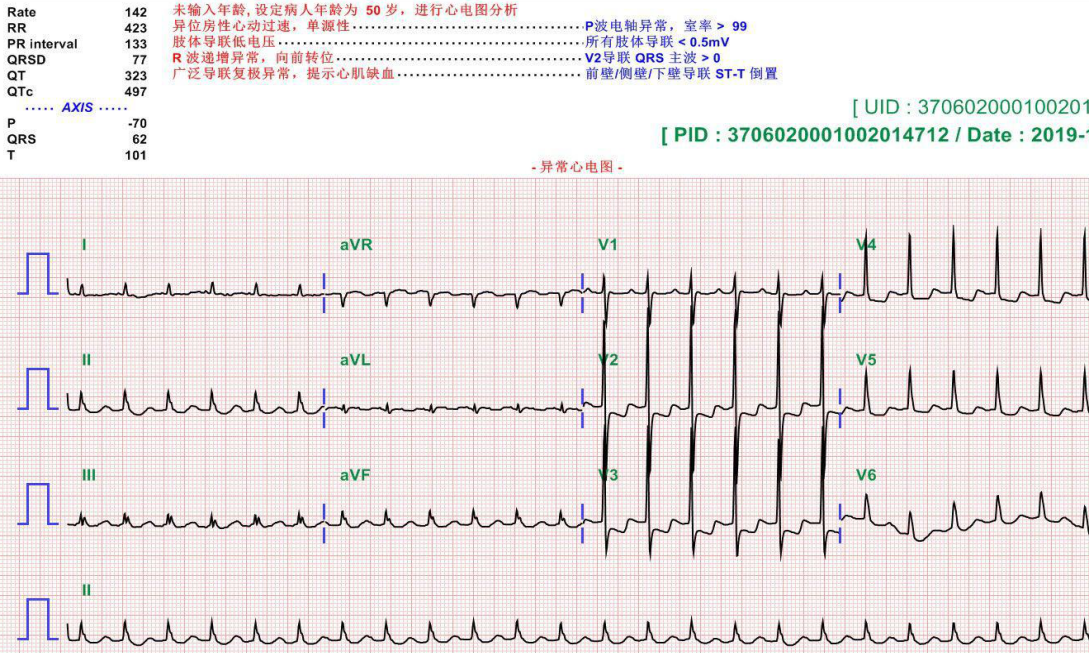


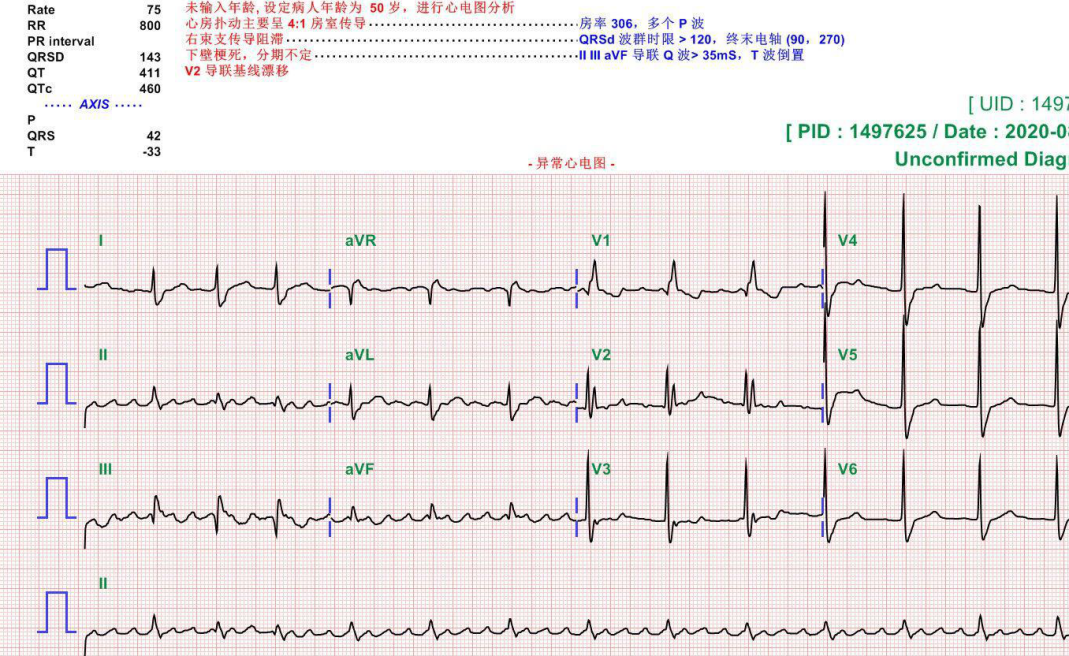


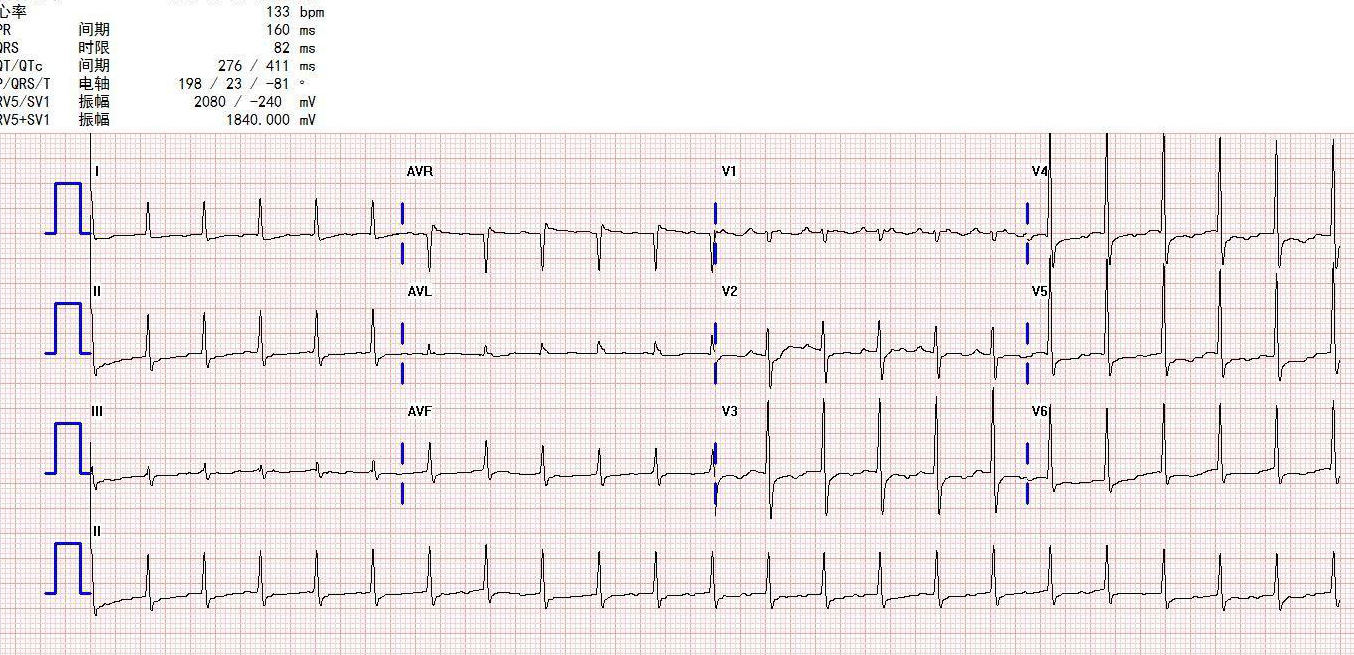


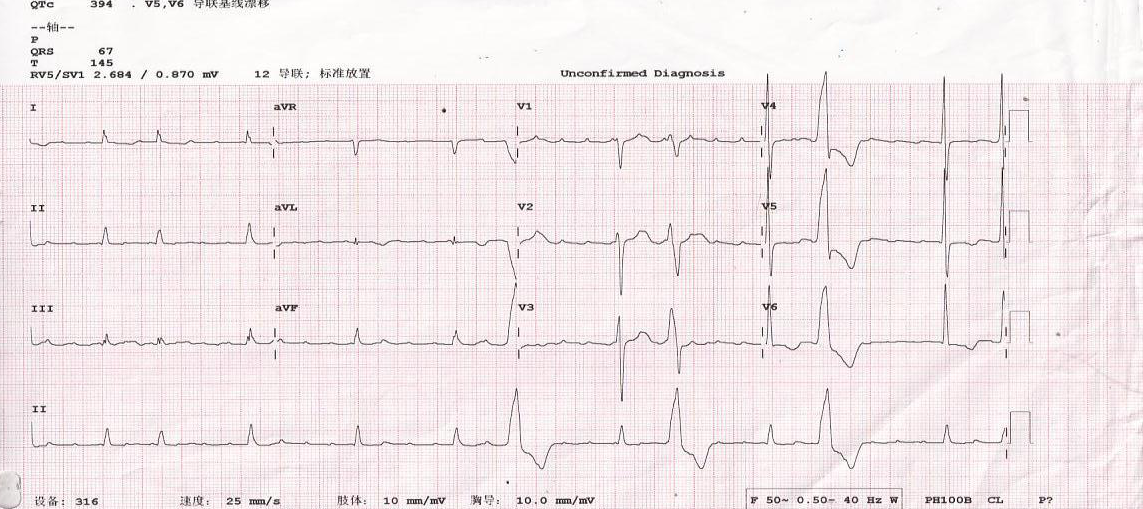

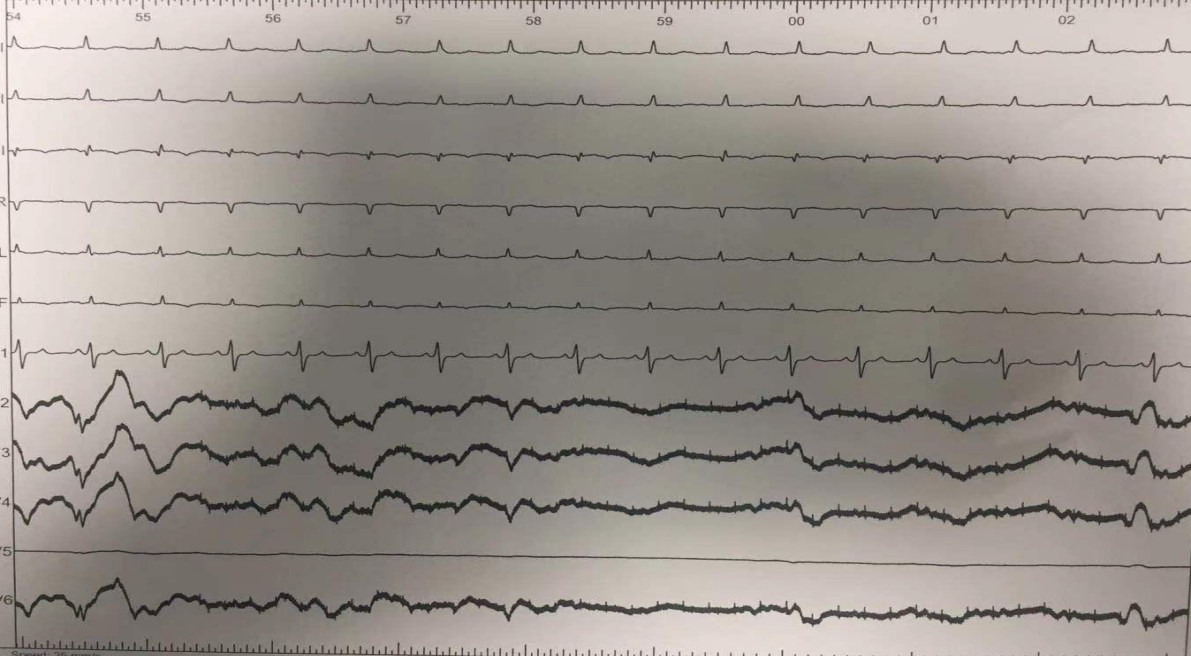

Supplement: Supplementary Materials — The Supplementary Material include Supplemental Method 1, Surface ECG of patients, Video 1, and Video 2. [file 2829070.f1.zip › 2829070.f1.docx]
